# The Predictive Value of Sepsis Scores for In-Hospital Mortality in Patients with Left-Sided Infective Endocarditis

**DOI:** 10.3390/tropicalmed9010023

**Published:** 2024-01-16

**Authors:** Bianca Leal de Almeida, Tania Mara Varejao Strabelli, Marcio Sommer Bittencourt, Vítor Falcão de Oliveira, Danielle Menosi Gualandro, Alfredo Jose Mansur, Flavio Tarasouchi, Lucas Pocebon, Milena Paixão, Flora Goldemberg, Reinaldo Salomão, Rinaldo Focaccia Siciliano

**Affiliations:** 1 Hospital das Clínicas, University of São Paulo Medical School, São Paulo 05403-010, SP, Brazil; vitorfalcaodeoliveira@gmail.com (V.F.d.O.); lz.pocebon@outlook.com (L.P.); rinaldo_focaccia@uol.com.br (R.F.S.); 2 Instituto do Coracao (InCor) do Hospital das Clinicas HCFMUSP, Faculdade de Medicina, Universidade de São Paulo, São Paulo 05403-000, SP, Brazil; tania.s@hc.fm.usp.br (T.M.V.S.); ajmansur@incor.usp.br (A.J.M.); milena.paixao@incor.br (M.P.); 3 Center for Clinical and Epidemiological Research, University Hospital, University of São Paulo, São Paulo 05508-010, SP, Brazil; 4 Hospital Israelita Albert Einstein, São Paulo 05653-000, SP, Brazil; 5 Escola Paulista de Medicina, Universidade Federal de São Paulo, São Paulo 04023-900, SP, Brazil; rsalomao@unifesp.br

**Keywords:** endocarditis, sepsis, SOFA, prognosis

## Abstract

Background: As infective endocarditis has particular characteristics compared to other infectious diseases, it is not clear if sepsis scores are reported with good accuracy in these patients. The aim of this study is to evaluate the accuracy of the qSOFA and SOFA scores to predict mortality in patients with infective endocarditis. Methods: Between January 2010 and June 2019, 867 patients with suspected left-sided endocarditis were evaluated; 517 were included with left-sided infective endocarditis defined as “possible” or “definite” endocarditis, according to the Modified Duke Criteria. ROC curves were constructed to assess the accuracy of qSOFA and SOFA sepsis scores for the prediction of in-hospital mortality. Results: The median age was 57 years, 65% were male, 435 (84%) had pre-existing heart valve disease, and the overall mortality was 28%. The most frequent etiologies were *Streptococcus* spp. (36%), *Enterococcus* spp. (10%), and *Staphylococcus aureus* (9%). The sepsis scores from the ROC curves used to predict in-hospital mortality were qSOFA 0.601 (CI95% 0.522–0.681) and SOFA score 0.679 (CI95% 0.602–0.756). A sub-group analysis in patients with and without pre-existing valve disease for SOFA ≥ 2 showed ROC curves of 0.627 (CI95% 0.563–0.690) and 0.775 (CI95% 0.594–0.956), respectively. Conclusions: qSOFA and SOFA scores were associated with increased in-hospital mortality in patients with infective endocarditis. However, as accuracy was relatively lower compared to other sites of bacterial infections, we believe that this score may have lower accuracy when predicting the prognosis of patients with IE, because, in this disease, the patient’s death may be more frequently linked to valvular and cardiac dysfunction, as well as embolic events, and less frequently directly associated with sepsis.

## 1. Introduction

The estimated incidence of infective endocarditis ranges from 2.4 to 11.6 per 100,000 persons per year [1,2,3]. Despite improvements in therapy, the case fatality rate in endocarditis patients is still high, ranging from 19 to 25% [4,5]. Cardiac complications and uncontrolled infection are the main causes for mortality in endocarditis patients [6].

Left-sided endocarditis encompasses the entire or partial involvement of the mitral and/or aortic valves, along with the adjacent anatomical structures [7]. This condition accounts for substantial systemic implications. On the other hand, isolated right IE is rare, and causes different manifestations, complications, and prognostics than left-sided IE; because of this, we decided to only study left-sided IE [4]. Therefore, patients with IE differ from patients with general bacterial infections; as a result, prognostic criteria employed in other infections may not be relevant to patients with endocarditis.

In 2016, the third sepsis consensus established new sepsis definitions, advocating for Sequential Organ Failure Assessment (SOFA) as a new predictor of mortality in patients with life-threatening infections (Sepsis-3) [8]. To improve the identification of potentially serious patients, quick-SOFA (qSOFA) was proposed as a bedside criterion, avoiding delays in sepsis diagnosis, starting early treatment, and aiming to reduce mortality caused by bacterial infections [9,10,11]. However, these scores have not been tested in a large sample of patients with infective endocarditis [12,13]. The aim of this study is to evaluate the accuracy of sepsis scores (qSOFA and SOFA) to predict in-hospital mortality in patients with left-sided endocarditis.

## 2. Materials and Methods

Patients with suspected left-sided infective endocarditis were admitted from January 2010 to June 2019 in the Instituto do Coracao (InCor) do Hospital das Clinicas HCFMUSP, Faculdade de Medicina, Universidade de Sao Paulo, Sao Paulo, SP, BR. InCor is a specialized tertiary teaching hospital focused on cardiovascular disease. We actively searched for suspected cases of left-sided infective endocarditis at wards and emergency units. This retrospective study reviewed the data of patients admitted to the emergency department with suspected left-sided infective endocarditis between January 2010 and Jane 2019. The cohort was extracted from a database of consecutively and prospectively selected patients with suspected infective endocarditis, as part of the Hospital Infection Control Unit surveillance activities during weekdays. The inclusion criterion was patients with suspected left-sided infective endocarditis who were over 18 years old. Suspected left-sided infective endocarditis was defined as the presence of at least one of the following criteria: (1) The presence of potential risk factors for infective endocarditis (mitral valve prolapse or degenerative valve disease with reflux on an echocardiogram, chronic rheumatic valve disease, valve prosthesis, congenital heart disease, a history of previous infective endocarditis, and intravenous drug use) and fever (>37.8 °C) or a clinical suspicion of systemic emboli or acute heart failure due to valve dysfunction; or (2) patients with fever (>37.8 °C) and a cardiac murmur or a clinical suspicion of systemic emboli and no other infection foci. Exclusion criteria were as follows: (i) patients who were classified as “rejected” for infective endocarditis, according to the Modified Duke Criteria [14] until discharge or death; and (ii) the use of intravenous antibiotics aimed at infective endocarditis etiology for more than 3 days before enrollment. The main predisposing factors for the occurrence of endocarditis are contained in the Modified Duke Criteria, published by Li et al. as “Proposed modifications to the Duke criteria for the diagnosis of infective endocarditis” in 2000 [14].

### 2.1. Studied Variables

We evaluated clinical and laboratory variables included in SOFA and qSOFA scores. To evaluate the SOFA score, we used clinical and laboratory data such as Glasgow Coma Scale, creatinine or urine output, blood pressure, platelets, PaO2/FIO2, and bilirubin levels. Septic shock (Sepsis-3) was defined as sepsis with persisting hypotension, requiring vasopressors to maintain adequate blood pressure and having a serum lactate level > 2 mmol/L (18 mg/dL), despite adequate volume resuscitation [8]. For the qSOFA, we collected the following data: respiratory rate, systolic blood pressure, and measurements from the Glasgow Coma Scale [8]. (Supplementary Material Table S1). The data were collected up to 3 days after the start of antibiotic therapy.

### 2.2. Definitions

Information from the Third International Consensus Definitions for Sepsis and Septic Shock (Sepsis-3) was used to evaluate qSOFA, SOFA, and septic shock [8] (Supplementary Material Table S1). According to this consensus, sepsis is defined as an acute change in the SOFA score, increasing by 2 or more points. For the qSOFA, at least 2 of the following clinical criteria together constitute a new bedside clinical score termed quick SOFA (qSOFA): respiratory rate of 22/min or greater and altered mentation, or systolic blood pressure of 100 mm Hg or less. Based on the sepsis-3 criteria, we considered the qSOFA ≥ 2 and SOFA ≥ 2 as the cutoff points for the prediction of in-hospital mortality in endocarditis patients [8].

Preexistent heart valve disease was defined in a patient if they had previously been admitted for IE and diagnosed with degenerative valve disease, rheumatic heart disease, mitral valve prolapse, bicuspid aortic valve, or congenital cyanotic heart disease.

All laboratory and clinical data, including the New York Heart Association classification (NYHA), systemic septic embolism, echocardiographic abscess, as well as the construction of sepsis scores/criteria were evaluated at the patient’s admission to the hospital.

The patients were systematically screened with blood cultures (Bactec^®^, Becton Dickson, Heidelberg, Germany) and an echocardiography. Indications for further diagnostic procedures, antibiotic treatment, and cardiac surgery were at the discretion of the treating physician, following the recommendations of the current IE guidelines [15].

For data collection and management, the Research Electronic Data Capture (REDCap) electronic system (REDCap 9.1.0—© 2019 Vanderbilt University, Nashville TN, USA) was used.

Lethality due to IE has been related to heart failure, systemic emboli, or septic shock. To analyze variables other than those that were sepsis-related, we performed univariate and multivariate analyses.

### 2.3. Clinical Endpoint

The primary endpoint was in-hospital death. All of the patients were followed until hospital discharge or death.

### 2.4. Statistical Analysis

Categorical variables comprised frequency and percentage; to test the association between categorical variables and hospital death outcome, we used the χ^2^ test or the Student’s *t*-test or exact Fisher test when appropriate. Continuous variables were presented as the median and were compared using the Mann–Whitney U test. Receiver operating characteristic curves (ROC) were constructed to assess the accuracy of sepsis scores for the prediction of in-hospital mortality. For multivariate analysis, variables with *p* < 0.1 in the univariate analysis were selected. The sensitivity, specificity, positive predictive value, and negative predictive values of the sepsis scores for the prediction of in-hospital death were evaluated. We also performed the same analysis for sub-groups with and without any known preexistent heart valve disease. Values of *p* < 0.05 indicate statistical significance.

Software STATA 14.0 (StataCorp. 2023. Stata Statistical Software: 14. College Station, TX, USA: StataCorp LLC.) was used for statistical analysis.

The ethics committee of Hospital das Clinicas HCFMUSP, Faculdade de Medicina, Universidade de Sao Paulo, Sao Paulo, SP, BR approved this study (with the approval number 0817/11).

## 3. Results

In this study, 867 patients with suspected infective endocarditis were evaluated; of these, 350 were excluded upon hospital admission (53 due to previous antibiotic use and 297 due to rejected IE), only 517 met the Duke Criteria for possible or confirmed IE, and the remaining patients were excluded from the study. Therefore, 517 patients met the standards of the Modified Duke Criteria for the diagnosis of infective endocarditis and were thus included (401 patients were classified as “defined”, and 116 were classified with “possible” infective endocarditis); therefore, we included as many patients with IE as was possible for this study and used the Duke Criteria to define them under the same analysis as the defined IE group.

Sepsis-3 was only published in 2016; thus, patients treated prior to this date did not always contain the clinical and laboratory parameters necessary to assess the SOFA and qSOFA in their initial assessment. Of the 517 patients enrolled in this study, 465 contained the necessary data needed to apply these scores at patient admission.

Both the baseline characteristics of patients with endocarditis and univariate analysis related to the outcomes are shown in Table 1. The majority were male (65%), and the median age was 57 years old (ranging from 18 to 87). Pre-existing heart valve disease was present in 84% (435): valve prosthesis—58% (303), rheumatic heart disease—33% (173), degenerative valve disease—17% (91), mitral valve prolapse—9% (49), bicuspid aortic valve—4% (21), and congenital cyanotic heart disease—4% (22).

As endocarditis complications, heart failure NYHA III/IV was present in 40%, valve abscesses were present in 12%, and systemic septic embolism was present in 19% (56% central nervous system, 35% spleen, and 14% limbs). The most common comorbidities were hypertension (52%) and diabetes mellitus (18.7%). It is important to note once more that InCor is a specialized tertiary teaching hospital focused on cardiovascular disease.

Of the 517 patients enrolled, 494 underwent echocardiography on admission; of these, 85% underwent transoesophageal echocardiography, and 15% underwent transthoracic echocardiography. We found that 57.6% of patients had vegetation, 12.5% had abscess, 7.2% had valve perforation, 44.5% had mitral insufficiency (moderate/severe), and 29.5% had aortic insufficiency (moderate/severe).

Pathogens were identified in 409 episodes (79.1%). The microorganisms most frequently isolated were *Streptococcus* spp. (36%), *Enterococcus* spp. (10%), and *Staphylococcus aureus* (9%). The other 124 microorganisms that were isolated were Gram-negative bacilli, fungi, and other atypical pathogens, all without statistical significance. Around 65% of *Staphylococcus aureus* were sensitive to methicillin; for the other microorganisms, we were unable to obtain a sensitivity profile.

**Figure 1:**
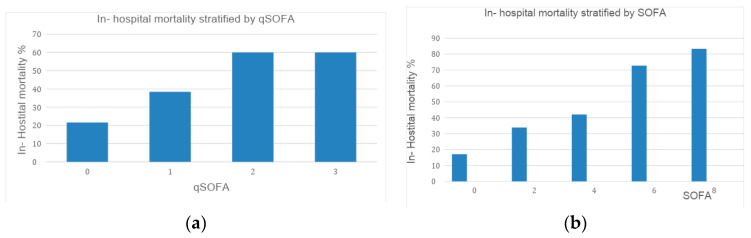
(**a**) In-hospital mortality stratified using qSOFA. The number of patients for each qSOFA variable: 0 (72); 1 (33); 2 (24); and 3 (3). (**b**) In-hospital mortality stratified using SOFA. The number of patients for each SOFA variable: 0 (29); 2 (21); 4 (8); 6 (8); and 8 (5).

Overall mortality was 28% (145/517), and 57% (272/517) needed heart valve surgery during hospitalization. qSOFA-stratified mortality and SOFA-stratified mortality are shown in Figure 1.

The median time between the onset of symptoms, diagnosis, and antibiotic therapy was 17 days—14 days for patients who died and 17 days for living patients, with no statistical difference in both. All of the diagnosed patients received antibiotic therapy on the same day; therefore, the median time between symptom onset, diagnosis, and symptom onset during antibiotic therapy was the same.

### 3.1. Survival and Non-Survival Groups in Comparison

Using univariate analysis, episodes of endocarditis presented higher mortality in patients with pre-existing hypertension, diabetes mellitus, and chronic kidney disease (Table 1). Conditions related to endocarditis, such as heart failure NYHA III/IV, systemic septic embolism, and *enterococci* e *staphylococci* etiologies, were associated with in-hospital death. At admission, laboratory analysis showed that non-survivor patients showed higher leucocytes, creatinine, and C-reactive protein, as well as lower hemoglobin and platelets counts (Table 1). 

qSOFA ≥ 2 and SOFA ≥ 2 were associated with in-hospital mortality (Table 2). As SOFA ≥ 2 showed better performance among sepsis scores, it was chosen for multivariate analysis. Other variables included in the multivariate analysis were hypertension, diabetes mellitus, chronic kidney disease, embolism, NYHA III/IV, *S. aureus* infection, age, C-reactive protein, hemoglobin, creatinine, and platelet count. In this analysis, the variables that remained associated with in-hospital death were the presence of diabetes mellitus and NYHA III/IV, SOFA ≥ 2, systemic septic embolism, and lower hemoglobin at admission (Table 3). The ROC curves of qSOFA and SOFA for the prediction of in-hospital mortality were 0.601 (CI95% 0.522–0.681) and 0.679 (CI95% 0.602–0.756), respectively. qSOFA ≥ 2 and SOFA ≥ 2 accuracy are presented at Table 4. Similar results were found when this prognostic analysis was made according to the Modified Duke Criteria: qSOFA ≥ 2 (ROC 0.545, CI95% 0.412–0.678) and SOFA ≥ 2 (ROC 0.667, CI95% 0.541- 0.792) for “Possible” endocarditis and qSOFA ≥ 2 (ROC 0.583, CI95% 0.515–0.651) and SOFA ≥ 2 (ROC 0.652, CI95% 0.589–0.716) for 401 “Definite” endocarditis episodes.

### 3.2. Comparison between Pre-Existing Valve Disease and No Pre-Existing Valve Disease

Patients without pre-existing valve disease were younger (52 vs. 58, *p* = 0.033), were more frequently on a hemodialysis regimen (17.1% vs. 4.6%, *p* < 0.001), had lower hemoglobin (9.9 vs. 11, *p* < 0.001), higher platelet count (227.500 vs. 191.000, *p* = 0.033), higher rate of embolization (39.5% vs. 14.8%, *p* < 0.001) and higher *S. aureus* etiology (18.3% vs. 7.8%, *p* = 0.003) than patients with pre-existing valve disease (Supplementary Material Table S2). The ROC curve to predict in-hospital mortality for qSOFA and SOFA scores among infective endocarditis patients with and without pre-existing valve disease was 0.627 and 0.775, respectively (Table 4). The accuracy for qSOFA ≥ 2 and SOFA ≥ 2 is presented in Supplementary Material Table S3. Considering that *S. aureus* is the most virulent microorganism in infective endocarditis, it could thus interfere with the application of the sepsis score; therefore, we performed SOFA score validation for predictive values according to this etiology: staphylococcal infections, qSOFA ≥ 2 (ROC 0.632, CI95% 0.456–0.807) and SOFA ≥ 2 (ROC 0.667, CI95% 0.501–0.832); non-staphylococcal infections, qSOFA ≥ 2 (ROC 0.563, CI95% 0.499–0.628) and SOFA ≥ 2 (ROC 0.645, CI95% 0.583–0.706).

## 4. Discussion

qSOFA and SOFA scores were directly related to outcomes in patients with infective endocarditis, even though they presented low accuracy overall in predicting all-cause in-hospital death. Interestingly, an increased SOFA score demonstrated better performance as a mortality predictor in patients without a pre-existing valve disease.

The Third International Consensus on Sepsis (Sepsis-3) emphasizes that sepsis is defined as a life-threatening organic dysfunction and can be identified with an acute change in a SOFA score of 2 or more; as this score reflects a mortality of approximately 10%, the authors emphasize that higher SOFA scores are associated with higher mortality. The qSOFA score was introduced as a possible predictive tool for bedside use to quickly identify a possible suspicion of infection. Although qSOFA is less robust than SOFA classification, it does not require laboratory testing and can be used for quick and repeated assessment; therefore, qSOFA criteria should be used to prompt clinicians to further investigate organ dysfunction and should not be used as a definitive classification tool [8].

For the main analyses, we used the Sepsis-3 definition of SOFA and qSOFA scores of 2 or more to evaluate prognosis; after all, this was the recommended value for defining sepsis and is widely used in clinical practice and in studies [8]. To demonstrate that the highest qSOFA and SOFA scores have an impact on greater mortality, as in other infections, we present stratified mortality data [16].

Since the publication of the Third International Sepsis Consensus (Sepsis-3) [8], several authors [9,16,17,18] have demonstrated the high accuracy of the SOFA score as a predictor of in-hospital mortality in patients with severe bacterial infections. Razani et al. [9]. studied Sepsis-3 score performance as a predictor of in-hospital death in 6024 patients with community-acquired pneumonia. They found ROC curves of 0.697 and 0.748 for the qSOFA and the SOFA, respectively. Additionally, *Songsangjida* and *Khawannimit* [18] evaluated its accuracy for mortality prediction concerning sepsis scores in 6968 patients in intensive care. They found the following ROC curve of the SOFA score in a sub-group of the patients: septic shock (Sepsis-3) (0.820), respiratory infection (0.847), and gastrointestinal infection (0.848).

Another report [16] evaluated 184,875 intensive care unit patients admitted with bacterial infections (gastrointestinal infection—10%, urinary tract infection—5%, and respiratory infection—18%) and showed that the SOFA had a higher accuracy for in-hospital mortality than the qSOFA (qSOFA ROC curve—0.607 vs. SOFA ROC curve—0.753) thus concluding that, among adults with suspected infections admitted to an ICU, an increase in the SOFA score of 2 or more had greater prognostic accuracy for in-hospital mortality than SIRS criteria or the qSOFA score, suggesting that SIRS criteria and qSOFA may have limited utility for predicting mortality in this population. For infections other than IE, it is already known that the SOFA and qSOFA have different performances for predicting sepsis and for the prognoses of these patients; therefore, we chose to present the performance of each of these scores in patients with IE.

There is little information related to the clinical use of sepsis scores as an early predictor of mortality in patients with infective endocarditis. Raith et al. [16] found that, in adults with suspected infections admitted to an ICU, an increase in the SOFA score had good prognostic accuracy for in-hospital mortality; on the other hand, we do not have much data for SOFA as a mortality predictor in patients with IE. We believe that this score may have a lower accuracy in predicting the prognosis of patients with IE because, in this disease, the patient’s death may be more frequently linked to valvular and cardiac dysfunction, as well as embolic events, and less frequently directly associated with sepsis [4].

Tamura et al. [19] found an association between qSOFA ≥ 2 and in-hospital adverse events (death, embolism, abscess, intracranial hemorrhage) in 83 patients with infective endocarditis. However, the sensitivity observed was 27%, and the specificity was 95%, similar to this study. Asai et al. [12], while studying the prognostic effect of sepsis scores in 66 cases of infective endocarditis, found that the SOFA ≥ 6 had high accuracy as an in-hospital death predictor (with an ROC curve of 0.915). Although the number of patients studied was small in this study [12], the high proportion of *staphylococcal* infections (53%) in this series may have influenced the SOFA score performance. Since *S. aureus* is known to be a highly virulent pathogen, an infection of this microorganism may be directly associated with sepsis complications [20].

This study verified the better prognostic performance of sepsis criteria in patients without pre-existing heart valve diseases. This population showed a higher prevalence of staphylococcal infections, as well as a higher rate of embolism at admission, which could be related to more susceptibility to complications associated with septicemia. An increased SOFA score by 2 or more points has shown promise as a mortality predictor in patients without pre-existing valve diseases, reaching closer accuracy to other bacterial infections. However, this has to be evaluated in a larger cohort.

Patients with pre-existing valvar diseases may have complications associated with valvular dysfunction rather than classic sepsis-related organic dysfunctions. Although an infection of *S. aureus* is an important risk factor for death [13,20,21,22], other prognostic factors are recognized in patients with endocarditis, such as advanced age [15,23,24], the pre-existence of chronic kidney disease [21,24,25], diabetes [1,22,26], prosthetic valve endocarditis [10,25,27,28], and heart failure [15,29,30]. Thus, it is possible that death due to endocarditis in patients with pre-existing heart valve diseases is more closely related to valve dysfunction or comorbidities than directly to sepsis complications [31]. Applying sepsis scores in patients with infective endocarditis on admission, as a SOFA ≥ 2, may help identify patients with an increased risk of death—mainly those without pre-existing heart valve diseases. However, this may lead to misleading risk stratification, particularly regarding patients with pre-existing heart valve diseases.

Some limitations should be considered. First, this is a single-center study in a cardiac referral hospital, which may reflect patients having a higher prevalence of valvular diseases and severe diseases, with higher mortality, than other general hospitals. In Brazil, we have a high rate of valvular diseases resulting from rheumatic etiology, which differs from countries with a high level of development; these facts may influence the results found here and hinder their applicability in different scenarios. The benefit of a SOFA score with an increase of 2 or more points as a prognostic marker in patients without any known pre-existing heart valve diseases needs to be confirmed in a larger cohort.

In August 2023, the new Infectious Endocarditis (IE) guideline from the European Society of Cardiology (ESC) was published [32], with several new features. This database and analysis were formed before this publication; therefore, all the clinical management and criteria followed previous guidelines [4].

## 5. Conclusions

qSOFA and SOFA scores were associated with increased in-hospital mortality in patients with infective endocarditis. However, as accuracy was relatively lower compared to other sites of bacterial infections, we believe that this score may have a lower accuracy in predicting the prognosis of patients with IE, because, in this disease, the patient’s death may be more frequently linked to valvular and cardiac dysfunction, as well as embolic events, and less frequently directly associated with sepsis. Additional studies are needed to determine a specific early prognostic score to predict mortality in these patients.

## Figures and Tables

**Table 1:** Univariate analysis for in-hospital mortality in patients with infective endocarditis.

Baseline Characteristic/ Variables	All Patients n = 517 (%)	Discharge n = 372 (%)	In-Hospital Death n = 145 (%)	OR	95% CI	*p* Value
Age (median; years)	57 (18–87)	56 (18–87)	58.7 (18–86)	-	-	-
Male	334 (64.6)	243 (65.3)	91 (62.7)	0.894	0.600–1.332	0.584
Hypertension	270 (52.2)	181 (48.6)	89 (61.3)	1.677	1.134–2.480	0.009
*Diabetes mellitus*	97 (18.7)	57 (15.3)	40 (27.5)	2.105	1.328–3.337	0.001
Chronic kidney disease	59 (11.4)	36 (9.6)	23 (15.8)	1.759	1.002–3.088	0.049
Hemodialysis	34 (6.5)	21 (5.6)	13 (8.9)	1.646	0.868–1.453	0.109
Pre-existing valve disease ^1^	435 (84.1)	315 (84.6)	120 (82.7)	0.868	0.801–3.382	0.536
*Staphylococcus aureus*	49 (9.4)	28 (7.5)	21 (14.4)	2.080	1.139–3.798	0.015
*Streptococcus group*	186 (35.9)	150 (40.3)	36 (24.8)	0.488	0.318–0.751	0.001
*Enterococcus species*	50 (9.6)	26 (6.9)	24 (16.5)	2.639	1.460–4.772	0.001
Culture positive	409 (79.1)	298 (80.1)	111 (76.5)	0.8107	0.511–1.285	0.372
NYHA III/IV	206 (39.8)	128 (34.4)	78 (53.7)	2.219	1.502–3.798	<0.001
Embolism (n = 514)	96 (18.6)	60 (16.2)	36 (24.8)	1.700	1.065–2.714	0.025
Valve abscesses (n = 505)	62 (12.2)	40 (10.9)	22 (15.6)	1.497	0.854–2.624	0.158
Hemoglobin (g/dL) (n = 500)	10.8 (4.5–23.8)	11 (5.3–23.8)	10.1 (4.5–16)	-	-	<0.001
Platelets (mg/L) (n = 498)	193.000 (2.000–557.000)	199.000 (2.000–550.000)	173.000 (12.330–557.000)	-	-	0.010
Leukocyte (mg/L) (n = 500)	10.360 (550–42.000)	9.960 (1.1000–35.000)	12.040 (550–42.000)	-	-	<0.001
C-reactive protein (mg/L) (n = 495)	89 (1.6–402)	86.6 (1.96–389.2)	141.5 (68.5–197)	-	-	0.012
Creatinine (mg/dL) (n = 499)	1.2 (0.45–15.4)	1.1 (0.45–15.5)	1.5 (0.53–12.34)	-	-	<0.001
Time of symptoms (n = 509)	16 (0–370)	17 (0–370)	14 (0–366)			0.264

CI = confidence interval; OR = Odds ratio; NYHA = New York Heart Association classification; ^1^ Pre-existing valve disease in patients at admission.

**Table 2:** Univariate analysis of Sepsis criteria/scores in patients with endocarditis.

Variables	All Patients n = 465 (%)	Discharge n = 372 (%)	In-Hospital Death n = 145 (%)	OR	95% CI	*p* Value
qSOFA ≥ 2	45 (9.6)	18(5.4)	27(20.4)	4.5	2.382–8.500	<0.001
SOFA ≥ 2	164 (35.2)	88(26.4)	76(57.5)	3.778	2.476–5.764	<0.001

CI = confidence interval; OR = Odds ratio; SIRS = Systemic inflammatory response syndrome.

**Table 3:** Multivariate analysis for predicting in-hospital mortality (n = 450).

Variable	OR	95% CI	*p* Value
NHYA III/IV	2.37	1.497–3.751	<0.001
Embolism at admission	1.91	1.097–3.324	0.022
SOFA ≥ 2	3.26	2.076–5.137	<0.001
Diabetes mellitus	2.45	1.490–4.345	0.001
Hemoglobin	0.860	0.773–0.956	0.005

CI = confidence interval; OR = Odds ratio; NYHA = New York Heart Association classification.

**Table 4:** qSOFA ≥ 2 and SOFA ≥ 2 accuracy for in-hospital mortality in patients with infective endocarditis.

Score	Sensitivity (95% CI)		Specificity (95% CI)	PPV (95% CI)		NPV (95%CI)	LR + (95% CI)
qSOFA ≥ 2	20 (14–27)		95 (92–97)	60 (46–74)		75 (71–79)	3.78 (2.16–6.63)
SOFA ≥ 2	58 (49–66)		74 (69–78)	46 (39–54)		81 (77–86)	2.18 (1.73–2.75)
		With pre-existing Valve disease **AUC ROC (95% CI)**			Without pre-existing valve disease **AUC ROC (95% CI)**		
qSOFA		0.551 (0.485–0.617)			0.718 (0.524–0.912)		
SOFA		0.627 (0.563–0.690)			0.775 (0.594–0.956)		

AUC ROC = area under the receiver operating characteristic curve; CI = confidence interval; PPV = positive predictive value; NPV = negative predictive value; LR+ = positive likelihood ratio.

## Data Availability

Data sharing is not applicable to this article.

## Supplementary Materials

The following supporting information can be downloaded at: https://www.mdpi.com/article/10.3390/tropicalmed9010023/s1, Table S1: qSOFA e SOFA scores; Table S2: Analysis and characteristics of patients with pre-existing valve disease and no pre-existing valve disease; Table S3: qSOFA ≥ 2 and SOFA ≥ 2 accuracy for in-hospital mortality in endocarditis patients with and without pre-existing valve disease.
