# Supplementary material for: The Predictive Value of Sepsis Scores for In-Hospital Mortality in Patients with Left-Sided Infective Endocarditis
Source: Trop Med Infect Dis. 2024 Jan 16;9(1):23. doi: 10.3390/tropicalmed9010023 (PMC10818832; doi:10.3390/tropicalmed9010023)
Supplement: Supplementary file 1 [file tropicalmed-09-00023-s001.zip › tropicalmed-2640868-supplementary.pdf]

Tropical Medicine and Infectious Disease

Supplementary Materials: The Predictive Value of Sepsis Scores for In-Hospital Mortality in Patients with Left-Sided Infective Endocarditis

Bianca Leal de Almeida [1,*], Tania Mara Varejao Strabelli [2], Marcio Sommer Bittencourt [3,4], Vítor Falcão de Oliveira [1], Danielle Menosi Gualandro [2], Alfredo Jose Mansur [2], Flavio Tarasouchi [2], Lucas Pocebon [1], Milena Paixão [2], Flora Goldemberg [1], Reinaldo Salomão [5] and Rinaldo Focaccia Siciliano [1,2]

Table S1. qSOFA e SOFA scores.

System/Score	0	1	2	3	4
**SOFA**					
Respiration PaO2/FIO2, mm Hg(kPa)	≥400 (53.3)	<400 (53.3)	<300 (40)	<200 (26.7) with respiratory support	<100 (13.3) with respiratory support
Coagulation Platelets,×1000/µL	≥150	<150	<100	<50	<20
Liver Bilirubin, mg/dL (µmol/L)	<1.2 (20)	1.2-1.9 (20-32)	2.0-5.9 (33-101)	6.0-11.9 (102-204)	>12.0 (204)
Cardiovascular MAP	≥70 mm Hg	<70 mm Hg	Dopamine <5 or dobutamine (any dose)*	Dopamine 5.1-15 or epinephrine ≤0.1 or norepinephrine ≤0.1*	Dopamine >15 or epinephrine >0.1 or norepinephrine >0.1*
Central nervous system (GCS)+	15	14-13	12-10	9-6	<6
Renal Creatinine, mg/dL(µmol/L) /Urine output, mL/d	<1.2 (110)	1.2-1.9 (110-170)	2.0-3.4 (171-299)	3.5-4.9 (300-440)/ <500	>5.0 (440)/ <200
**qSOFA**					
Respiratory rate >22/min<br>Altered mentation<br>Systolic blood pressure<100mmHg					

Consensus Definitions for Sepsis and Septic Shock, *JAMA* February 23, 2016 Volume 315, Number 8.

+= Glasgow Coma Scale; *= Catecholamine doses are given as µg/kg/min for at least 1 hour, MAP= mean arterial pressure

Table S2. Analysis and characteristics of patients with pre- existing Valve disease and no pre- existing valve disease.

Characteristic/ Variables	Pre- existing Valve disease n= 435 (%)	Non Pre- existing Valve disease n=82 (%)	OR	95%CI	P value
**Baseline characteristics**					
Age (median; years)	58(18- 87)	52 (20-85)	-	-	0.033
Male	275(63.2)	59 (72)	0.670	0.398 - 1.126	0.129
Hypertension	235(54)	35(42.7)	1.577	0.979 – 2.541	0.060
Diabetes mellitus	79(18.2)	18(22)	0.780	0.443 – 1.404	0.420
Chronic kidney disease	52(12)	7(8.5)	0.789	0.443 – 1.404	0.037
Hemodialysis	20(4.6)	14(17.1)	0.234	0.112 – 0.485	<0.001
NYHA III/IV	176(40.5)	30(36.6)	1.117	0.722 – 1.910	0.511
qSOFA ≥ 2 (n=392)	33(8.4)	12(16.4)	0.467	0.228 – 0.954	0.033
SOFA ≥ 2 (n=392)	135(34.4)	29(39.7)	0.797	0.477 - 1.331	0.385
S. aureus	34(7.8)	15(18.3)	0.378	0.195 – 0.733	0.003
Streptococcus group	160(36.8)	26(31.7)	1.253	0.756 – 2.075	0.380
Enterococcus species	44(10.1)	6(7.3)	1.425	0.586 – 3.462	0.432
Culture positive	347(79.8)	62(75.6)	1.272	0.729 – 2.217	0.395
Embolism * (N=433)	64(14.8)	32(39.5)	0.265	0.158 – 0.446	<0.001
Valve abscesses + (N=426)	51(12)	11(13.9)	0.840	0.417 – 1.694	0.627
Death	120(27.6)	25(30.5)	0.868	0.518 – 1.453	0.592
**Lab values at admission(median)**					
Hemoglobin (g/dL) (n=427)	11 (4.5-20.6)	9.9 (5-23.8)	-	-	<0.001
Platelets (mg/L) (n=426)	191.000 (2.000-557.000)	227.500 (123.000-481.000)	-	-	0.033
Leukocyte (mg/L) (n=427)	10.540 (11.000-40.470)	9.600 (550- 42.000)	-	-	0.356
C-reactive protein (mg/L) (n=423)	89 (1.96-402)	90 (6.24-389.2)	-	-	0.259
Creatinine (mg/dL) (n=426)	1.19 (0.45-13.1)	1.31(0.63-15.54)	-	-	0.176

Number (%); CI= confidence interval; OR= Odds ratio; NYHA= New York Heart Association classification; *= suspect embolism in admission; += valve abscesses in the admission.

Table S3. qSOFA ≥ 2 and SOFA ≥ 2 accuracy for in-hospital mortality in endocarditis patients with and without pre-existing valve dis.ease

Score	Sensitivity (95% CI)	Specificity (95% CI)	PPV (95% CI)	NPV (95%CI)	LR + (95% CI)
**With pre-existing valve disease.**					
**qSOFA ≥ 2**	**17(11 – 25)**	**94(91 – 96)**	**53(38 – 68)**	**75(71 – 79)**	**3.1(1.78 – 5.68)**
SOFA ≥ 2	55(46 – 63)	71(61 – 76)	41(34 – 49)	81(76 – 85)	1.9(1.51 – 2.43)
**Without pre-existing valve disease.**					
**qSOFA ≥ 2**	**41.7(24 – 61)**	**95(86 – 98)**	**83(55 – 95)**	**77(65 – 85)**	**10.1(2.45 – 43)**
SOFA ≥ 2	79(59 – 90)	79(64 – 85)	65(47 – 80)	88(76 – 95)	3.8(2.15 – 6.99)

CI = confidence interval; PPV = positive predictive value; NPV = negative predictive value; LR+ = positive likelihood ratio
